# Combined effects of FTO rs9939609 and MC4R rs17782313 on elevated nocturnal blood pressure in the Chinese Han population

**DOI:** 10.5830/CVJA-2015-064

**Published:** 2016

**Authors:** Yanlei Sun, Wu Jun, Jiazhong Sun, Mei Yang

**Affiliations:** Department of Endocrinology, the Third Hospital of Wuhan, Wuhan, China, 430060; Department of Endocrinology, the Third Hospital of Wuhan, Wuhan, China, 430060; Department of Endocrinology, Zhongnan Hospital, Wuhan University, Wuhan, China, 430071; Department of Endocrinology, Zhongnan Hospital, Wuhan University, Wuhan, China, 430071

**Keywords:** gene polymorphism, daytime blood pressure, nocturnal blood pressure, Chinese Han population

## Abstract

**Aim:**

In this study we investigated the association of *FTO* rs9939609 and *MC4R*. rs17782313 with elevated blood pressure in the Chinese Han population, and analysed the relationship between the rs9939609 and rs17782313 variants.

**Methods:**

We tested the rs9939609 and rs17782313 variants with the sequence-retrieval method.

**Results:**

The increase in odds ratios of the A allele of rs9939609 and the C allele of rs17782313 for nocturnal blood pressure were 1.37 and 1.69. The nocturnal blood pressure of participants simultaneously carrying the A and C alleles was significantly higher than the blood pressure of those carrying neither *FTO* nor *MC4R* risk alleles (*p* < 0.05), and that of the controls carrying only the A or C alleles (*p* < 0.05). No association between the *FTO* or *MC4R* genes with daytime hypertension was found in this Chinese population (*p* > 0.05).

**Conclusion:**

Our data suggest that the rs9939609 and rs17782313 variants may be significantly associated with nocturnal but not daytime blood pressure levels and their combined effects were significant in this Chinese Han population.

Elevated blood pressure increases the risk of experiencing cardiovascular events such as myocardial infarction and stroke. Current observational data suggest that body mass index (BMI) may have a causal role in the aetiology of hypertension, but this may be influenced by confounding and reverse causation.^1^

Genetic factors play an important role in the development of hypertension. Recent studies have revealed a strong association between common variants in introductory studies on the *FTO* gene and obesity in children and adults.^2^-^4^ Frayling and co-workers found that each rs9939609 allele (chr16:52,378,028; dbSNP build 129) increased body weight by 1.2 kg in the general adult population and conferred a 31% higher risk of developing obesity.^5^

*FTO* protein is a key link between the central nervous system and energy balance. It was found to be ubiquitously expressed in the hypothalamus and is thought to mediate this effect through its influence on energy homeostasis. The hypothalamus, however, also regulates blood pressure.^6^ Masked (nocturnal) hypertension is common in patients with type 2 diabetes mellitus.^6^ Masked hypertension (normal blood pressure in the clinic but elevated levels when measured outside the clinic) is associated with an increased risk of cardiovascular disease. Therefore, we investigated whether the *FTO* risk variant may not be associated only with obesity and BMI, but also with elevated nocturnal blood pressure.

The *MC4R* gene, encoding for the melanocortin 4 receptor, was the first locus at which mutations were associated with dominantly inherited morbid human obesity, and was the commonest genetic cause of human obesity.^7^ The rs17782313 C allele (chr18:56,002,077; dbSNP build 129), located 188 kb downstream of *MC4R*, was similarly associated with obesity [OR = 1.30 (1.20–1.41)] in populations of European origin.^8^ Cardiovascular risk factors such as type 2 diabetes mellitus,^9^,^10^ insulin resistance,^11^ and hypertension^12^ were associated with the risk allele A for *FTO* rs9939609 and the risk allele C for *MC4R* rs17782313, regardless of BMI.^9^,^10^ In Marcadenti and colleagues’ study, however, common genetic variants of *FTO* rs9939609 had a positive association with BMI and neck circumference, and *MC4R* rs17782313 in women, but a negative association with diastolic and mean blood pressure in hypertensive men in southern Brazil.^13^

In the present study, we investigated the association of *FTO* and *MC4R* gene polymorphisms with hypertension in the Chinese Han population and analysed the relationship between *FTO* rs9939609 and *MC4R* rs17782313 variants.

## Methods

The subjects were divided into two groups comprising a daytime hypertension group (575 patients) and a night-time hypertension group (583 patients). The number of control subjects was 1 200. We recruited by physical examination 2 358 non-related individuals, aged 50 to 70 years (1 175 men and 1 183 women), who all belong to the Han nationality from the Hubei province of China.

The study was carried out in the examination centre at the ZhongNan Hospital of Wuhan University. Daytime normotension was defined as daytime blood pressure < 135/85 mmHg.^6^ Night-time was defined as the time from when the patient went to bed until when the patient got out of bed the following morning. Nocturnal normotension was defined as night-time blood pressure < 120/70 mmHg.^6^ The individuals were selected for the daytime and nocturnal hypertension groups on the basis of having blood pressure levels ≥ 135/85 mmHg or ≥ 120/70 mmHg, respectively. The control group of 1 200 subjects had normal clinical and biochemical characteristics.

A selection criterion was that these subjects were not on any hypotensive drugs or had stopped taking the drugs a week earlier. Additional selection criteria were the absence of (1) secondary hypertension, (2) diastolic blood pressure (DBP) 110 mmHg on blood pressure-lowering medication, (3) gross obesity (BMI > 35 kg/m^2^), (4) diabetes mellitus, (5) renal dysfunction (serum creatinine > 180 mmol/l), (6) liver disease, (7) severe physical or mental disease (for example, malignancy, terminal cancer or dementia), (8) pregnancy, and (9) substance abuse, including alcohol.

Clinic normotension was defined as blood pressure < 130/80 mmHg with or without blood pressure-lowering medications. Clinic-measured blood pressure (clinic BP) was the average of three seated measurements taken one minute apart by specially trained nurses. Ambulatory BP measurement devices (Spacelab 90217, Spacelabs and Redmond, WA, USA) were set to measure the BP at 20-minute intervals for 24 hours.

Each subject donated 5 ml of blood for genomic DNA extraction. For genotyping procedures in our study, refer to a previous report.^14^ The polymerase chain reaction (PCR) was performed on an automated DNA thermal cycler (Beijing Institute of Technology, China) with the primers of FTO rs9939609 (FP:5′- AAGAGATGATCTCAAATCTACTTTATGAGATA-3′ and RP:5′-TTAGAGTAACAGAGACTATCCAAGTGCATCAT-3′, annealing temperature 54°C, 30 cycles and a 155 bp product).^6^ The primers of *MC4R* rs17782313 were designed using the primer 5 software (FP: 5′-AGGA AACAGCAGGGATAGGG-3′ and RP:5′-TGCTGAGACAGGTTCAT AAAAAG-3′, annealing temperature 56°C, 30 cycles and a 407 bp product). The *MC4R* rs17782313 and *FTO* rs9939609 variants were genotyped using sequence retrieval (SinoGenoMax Co, Ltd).

## Statistical analysis

Statistical analysis was performed using SPSS 11.5 for Windows. Genotype and allele frequencies were compared with the Hardy–Weinberg equilibrium model and then analysed using chi-squared testing and contingency tables, respectively. Allelic and genotypic associations of the *FTO* rs9939609 and *MC4R* rs17782313 variants that were found to be significant were evaluated by computing odds ratios and 95% confidence intervals (CI). All data were presented as means ± SD. The clinical and biochemical characteristics between these genotypes were compared by one-way ANOVA; *p* < 0.05 was considered significant. All analyses were adjusted for gender, age and geographical region.

## Results

The basic characteristics of the participants are shown in Table 1.

**Table 1:** Characteristics of the study sample

*Parameters*	*Daytime hypertension (n = 575)*	*Nocturnal hypertension (n = 583)*	*Controls (n = 1200)*
Age (years)	56.9 ± 12.8	57.70±13.7	56.7 ± 8.7
Clinic SBP (mmHg)	159.5 ± 4.5	130.9±4.4	126.8 ± 4.3
Clinic DBP (mmHg)	93.8 ± 4.3	85.7 ± 4.6	77.9 ± 4.8
Nocturnal SBP (mmHg)	109.3 ± 9.8	130.4 ± 9.2	100.8 ± 6.5
Nocturnal DBP (mmHg)	61.7 ± 7.6	79.5 ± 7.5	59.5 ± 7.5
Daytime SBP (mmHg)	155.7 ± 5.0	129.1 ± 5.7	120.2 ± 6.4
Daytime DBP (mmHg)	96.9 ± 6.6	81.5 ± 4.2	75.6 ± 6.1
hs-CRP (mg/l)	0.68 (0.52–0.75)	0.72 (0.67–0.80)	0.57 (0.49–0.65)
TC (mmol/l)	4.31 ± 1.20	4.32 ± 1.61	4.13 ± 1.65
TG (mmol/l)	2.15 ± 1.37	2.18 ± 1.51	2.20 ± 1.58
LDL cholesterol (mmol/l)	3.05 ± 1.98	3.03 ± 1.96	2.92 ± 1.93

SBP: systolic blood pressure, DBP: diastolic blood pressure, hs-CRP: highsensitivity C-reactive protein, TC: total cholesterol, TG: triglycerides, LDL: low-density lipoprotein. Data are means ± SD, median (interquartile range) or percentages unless otherwise indicated.

## Effects of FTO and MC4R on daytime hypertension

The effects of *MC4R* and *FTO* on daytime hypertension were first investigated independently of each other. All the genotype and allele frequencies of *FTO* rs9939609 and *MC4R* rs17782313 were in Hardy–Weinberg equilibrium (*p* > 0.05). The frequencies are presented in Table 2.

**Table 2:** Patient characteristics and demographics

		*FTO rs9939609*					*MC4R rs17782313*				
		*Genotypes, n (frequency)*			*Alleles, n (frequency)*		*Genotypes, n (frequency)*			*Alleles, n (frequency)*	
*Groups*	n	*AA*	*AT*	*TT*	*A*	*T*	*CC*	*CT*	*TT*	*C*	*T*
Daytime hypertension	575	96 (16.7)	258 (44.9)	221 (38.4)	450 (39.1)	700 (60.9)	49 (8.5)	230 (40.0)	296 (51.5)	328 (28.5)	822 (71.5)
Controls	1200	205 (17.1)	545 (45.4)	450 (37.5)	955 (39.8)	1445 (60.2)	116 (9.7)	476 (39.7)	608 (50.6)	708 (29.5)	1692 (70.5)

Data are means (SD) for genotypic classes in unrelated individuals.

*FTO* rs9939309: with Pearson χ^2^ test, comparison of genotypes: daytime hypertension vs controls, χ^2^ = 0.18, *p* > 0.05; comparison of alleles: daytime hypertension vs controls, χ^2^ = 0.03, *p* > 0.05;

*MC4R* rs17782313: with Pearson χ^2^ test, comparison of genotypes: daytime hypertension vs controls, χ^2^ = 0.90, *p*. > 0.05; comparison of alleles: daytime hypertension vs controls, χ^2^ = 0. 41, *p* > 0.05.

The effects of *FTO* rs9939609 on daytime hypertension are shown in Table 2. All frequencies are presented in Table 2. We found no significant association between the FTO gene and the Chinese Han population with regard to daytime hypertension (*p* > 0.05). The A allele frequency and the AA frequencies were not obviously different between the daytime hypertension group of patients and the controls.

The effects of *MC4R* rs17782313 on daytime hypertension are given in Table 2. No significant association was observed between *MC4R* and the Chinese Han population with regard to daytime hypertension (*p* > 0.05). The C allele frequency and the CC frequencies were not obviously different between the daytime hypertension group of patients and the controls.

## Effects of FTO and MC4R on nocturnal hypertension

The effects of *FTO* rs9939609 on nocturnal hypertension are shown in Table 3. Interestingly, the distribution of the genotypes and the two alleles were significantly different between the nocturnal hypertension group of patients and the controls (χ^2^ = 18.54 and χ^2^ = 19.39, respectively; *p* < 0.05). The A allele frequency and the AA frequencies were significantly higher in the patients than in the controls, as seen in Table 3. The increase in odds ratio of the A allele for the nocturnal blood pressure group was 1.37 (95% CI: 1.19–1.58). The genotypic odds ratio for elevated nocturnal blood pressure was 1.82 (95% CI: 1.38–2.41) for the AA genotype, and 1.39 (95% CI: 1.10–1.75) for the AT genotype.

**Table 3:** FTO rs9939609 and MC4R rs17782313 distributions in nocturnal hypertension and control groups

		*FTO rs9939609*					*MC4R rs17782313*				
		*Genotypes, n (frequency)*			*Alleles, n (frequency)*		*Genotypes, n (frequency)*			*Alleles, n (frequency)*	
*Groups*	n	*AA*	*AT*	*TT*	*A*	*T*	*CC*	*CT*	*TT*	*C*	*T*
Nocturnal hypertension	583	140 (24.0)	281 (48.2)	162 (27.8)	561 (48.1)	605 (51.9)	82 (14.1)	252 (43.2)	249 (42.7)	416 (35.7)	750 (64.3)
Controls	1200	205 (17.1)	545 (45.4)	450 (47.5)	955 (39.8)	1445 (60.2)	116 (9.7)	476 (39.7)	608 (50.6)	708 (29.5)	1692 (70.5)

Data are means (SD) for genotypic classes on unrelated individuals.

*FTO* rs9939309: with Pearson χ^2^ test, comparison of genotypes: nocturnal hypertension vs controls, χ^2^ = 18.54, *p* < 0.05; comparison of alleles: nocturnal hypertension vs controls, χ^2^ = 19.39, *p* < 0.05;

*MC4R* rs17782313: with Pearson χ^2^ test, comparison of genotypes: nocturnal hypertension vs controls, χ^2^ = 15.21, *p* < 0.05; comparison of alleles: nocturnal hypertension vs controls, χ^2^ = 12.88, *p* < 0.05.

The effects of *MC4R* rs17782313 on nocturnal hypertension is shown in Table 3. The distribution of the genotype and the two alleles were also significantly different between the nocturnal hypertension group and the controls (χ^2^ = 15.21 and χ^2^ = 12.88, respectively; *p* < 0.05). The C allele frequency and CC frequencies were significantly higher in the patients than in the controls, as seen in Table 3. The increase in odds ratio of the C allele for the nocturnal hypertension group was 1.69 (95% CI: 1.31–3.32). The genotypic odds ratio for nocturnal hypertension was 1.54 (95% CI: 1.39–4.13) for the CC genotype, and 1.28 (95% CI: 1.60–2.88) for the CT genotype.

**Fig. 1.**
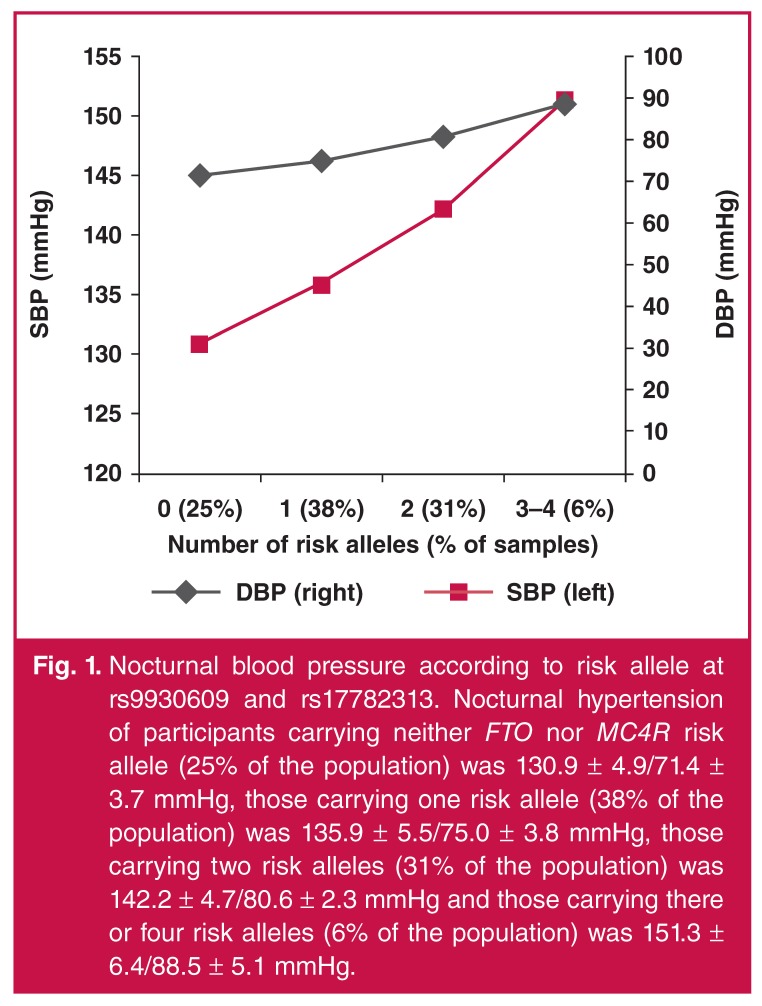
Nocturnal blood pressure according to risk allele at rs9930609 and rs17782313. Nocturnal hypertension of participants carrying neither *FTO* nor *MC4R* risk allele (25% of the population) was 130.9 ± 4.9/71.4 ± 3.7 mmHg, those carrying one risk allele (38% of the population) was 135.9 ± 5.5/75.0 ± 3.8 mmHg, those carrying two risk alleles (31% of the population) was 142.2 ± 4.7/80.6 ± 2.3 mmHg and those carrying there or four risk alleles (6% of the population) was 151.3 ± 6.4/88.5 ± 5.1 mmHg.

The combined effects of *FTO* rs9939609 and *MC4R* rs17782313 on nocturnal hypertension is shown in Fig. 1. In this study, we observed that the nocturnal blood pressure of the participants simultaneously carrying the A and C alleles was significantly higher than the BP of those carrying neither *FTO* nor *MC4R* risk allele (χ^2^ = 28.79, *p* < 0.05), and the BP of the controls carrying only the A or C alleles (χ^2^ = 25.74, *p* < 0.05) (Fig. 1).

## Discussion

Obesity and BMI are known to be associated with hypertension. Increases in BMI lead to an increase in the burden of hypertension. However, Ernsberger and Haskew found increasing prevalence of obesity as well as average BMI levels were accompanied by significant decreases in blood pressure level and prevalence of hypertension.^15^ This has led to questions about the nature of the association between obesity and hypertension.

Gregg *et al.* found both nocturnal and daytime systolic blood pressure predicted cardiovascular events independently of clinic systolic BP levels.^16^ In the general population, Ernsberger and Haskew found nocturnal BP was a better predictor of fatal cardiovascular events than daytime BP.^15^ Troiano and co-workers found the risk for cardiovascular death increased more steeply with increasing nocturnal BP levels than with increasing daytime BP levels.^17^

In our study, the analysis demonstrated a significant association of the *FTO* and *MC4R* genes with nocturnal blood pressure in the Chinese Han population (*p* < 0.05). The combined effects of *FTO* and *MC4R* played an important role in nocturnal blood pressure levels in this population. Nocturnal blood pressure levels of the participants carrying three or four risk alleles were higher than those with neither *FTO* nor *MC4R* risk allele (*p* = 0.008), those with one risk allele (p = 0.025), and those with two risk alleles (*p* = 0.041). The mechanism may be related to the *FTO* protein, which is a key link between the central nervous system and energy balance.

The *FTO* gene function is unknown but based on its predicted structure, the *FTO* gene encodes for a non-haeme (FeII) dioxygenase with a potential role in adaptation to hypoxia, lipolysis or DNA methylation.^17^,^18^ The *FTO* protein is expressed in almost all tissues; at the cellular level it has a nuclear localisation.^17^ The molecular mechanisms involved in the pathogenesis of obesity as well as the role of *FTO* gene in other complex disorders are unknown.

Various studies have shown cardiovascular risk factors such as type 2 diabetes mellitus,^9^,^10^ insulin resistance,^11^ and hypertension^12^ were associated with the risk allele A for *FTO* rs9939609 and the risk allele C for *MC4R* rs17782313, regardless of BMI.^9^,^10^ But in Marcadenti and colleagues’ study, common genetic variants of *FTO* rs9939609 had positive associations with BMI and neck circumference and *MC4R* rs17782313 in women, but a negative association with diastolic and mean blood pressure in hypertensive men in southern Brazil.^13^ However, these associations need to be confirmed by further replication studies, particularly in other ethnic populations. Brazilians and Chinese are different in their environmental risk factors, medical profiles, body composition and genetic backgrounds.

This study has some limitations that should be taken into account when interpreting the results. The analysis of genotype by gender was exploratory and it was underpowered to detect small differences. Therefore, some associations that achieved statistical significance in the overall analysis remained only as a trend towards that association. The association of gene variants with anthropometric indices should be confirmed in further studies with statistical power to carry out analysis by gender.

## Conclusion

We found that the *FTO* and *MC4R* genes were risk factors for nocturnal hypertension in this Chinese Han population, and their combined effects played an important role in nocturnal hypertension. However, even if a gene were considered associated with hypertension in certain populations, to expand the conclusion to all human populations is arbitrary. Furthermore, in the investigation of hypertension, obesity or diabetes, not only genetic factors but other factors, such as environment or geographic location could play a role. All these factors could have different effects on obesity or diabetes and they could impact on each other. Therefore whether or how a single gene could be associated with nocturnal hypertension is a complicated question. To decipher this, one would need long-term studies with numerous patients. *FTO* is a new gene reported by Yi-Cheng and co-workers in 2007,^2^ and thus far we have not answered all the questions since there has been minimal research on this gene among the different nations. We need more studies, since from a population perspective, only the combined effect of the most potent genetic variants should be considered.
